# Doppler-Derived Renal Functional Reserve in the Prediction of Postoperative Acute Kidney Injury in Patients Undergoing Robotic Surgery

**DOI:** 10.1213/ANE.0000000000006967

**Published:** 2024-06-17

**Authors:** Gianluca Villa, Sara Samoni, Mirko Muzzi, Sergio Fabbri, Faeq Husain-Syed, Lorenzo Tofani, Marco Allinovi, Laura Paparella, Pietro Spatafora, Roberto Di Costanzo, Zaccaria Ricci, Sergio Serni, Stefano Romagnoli

**Affiliations:** From the * Department of Health Sciences, Section of Anesthesiology, and Intensive Care, University of Florence, Florence, Italy; † Department of Anesthesia and Intensive Care, Azienda Ospedaliero Universitaria Careggi, Florence, Italy; ‡ Unit of Nephrology, Dialysis and Renal Transplant, Medical Department, Fondazione IRCCS Ca’ Granda Ospedale Maggiore Policlinico, Milan, Italy; § Department of Internal Medicine II, Division of Nephrology, University Hospital Giessen and Marburg, Justus-Liebig-University Giessen, Giessen, Germany; ‖ Department of statistics, informatics, applications, University of Florence, Florence, Italy; ¶ Unit of Nephrology, Dialysis and Transplantation, Geriatric Department, Careggi Hospital, University of Florence, Florence, Italy; # Department of Experimental and Clinical Medicine, University of Florence, Florence, Italy; ** Unit of Urological Minimally, Invasive Robotic Surgery and Renal Transplantation, Department of Oncology and Robotic Surgery, Careggi Hospital, University of Florence, Florence, Italy; †† Pediatric Intensive Care Unit, Department of Anesthesia and Critical Care, Meyer Children’s University Hospital, IRCCS, Florence, Italy.

## Abstract

**BACKGROUND:**

Postoperative acute kidney injury (PO-AKI) is a frequent complication after surgery. Various tools have been proposed to identify patients at high risk for AKI, including preoperative serum creatinine or estimated glomerular filtration rate (eGFR), urinary cell cycle arrest, and tubular damage biomarkers; however, none of these can appropriately assess AKI risk before surgery. Renal functional reserve (RFR) screened by the Doppler-derived intraparenchymal renal resistive index variation (IRRIV) test has been proposed to identify patients at risk for AKI before a kidney insult. IRRIV test has been developed in healthy individuals and previously investigated in cardiac surgery patients. This study aims to evaluate the value of the IRRIV test in identifying PO-AKI among patients undergoing robotic abdominal surgery in the Trendelenburg position for pelvic oncological disease.

**METHODS:**

We performed a prospective, double-blinded, observational study. Preoperative baseline renal function and RFR were assessed in 53 patients with baseline eGFR >60 mL/min/1.73 m^2^, undergoing robotic surgery in the Trendelenburg position for pelvic oncological disease. The capability of Doppler-derived RFR in predicting PO-AKI was investigated with the area under the receiver operating characteristic curve (ROC-AUC).

**RESULTS:**

Approximately 15.1% of patients developed AKI within the first 3 postoperative days. Thirty-one (58.5%) patients had a physiologic delta-RRI (ie, ≥0.05), while 22 (41.5%) patients did not. The ROC-AUC for PO-AKI was 0.85 (95% confidence interval [CI], 0.74–0.97; *P* = .007) for serum creatinine, 0.84 (95% CI, 0.71–0.96; *P* = .006) for eGFR, and 0.84 (95% CI, 0.78–0.91; *P* = .017) for delta-RRI. When combined with eGFR, the ROC-AUC for delta-RRI was 0.95 (95% CI, 0.9–1).

**CONCLUSIONS:**

Our findings show that the preoperative assessment of Doppler-derived RFR combined with baseline renal function improves the capability of identifying patients at high risk for PO-AKI with eGFR >60 mL/min/1.73 m^2^ after robotic abdominal surgery in Trendelenburg position for pelvic oncological disease.

**KEY POINTS**

**Question:** Among patients with apparently normal kidney function (ie, estimated glomerular filtration rate [eGFR] >60 mL/min/1.73 m^2^) undergoing robotic surgery for pelvic oncological disease, can we preoperatively identify patients at high risk for postoperative acute kidney injury (PO-AKI)?

**Findings:** Combination of the preoperative assessment of Doppler-derived renal functional reserve (RFR) and the baseline renal function (eg, serum creatinine or eGFR) seems to improve the capability of identifying patients at high risk for PO-AKI among those with apparently normal preoperative kidney function.

**Meaning:** Doppler-derived RFR assessed by intraparenchymal renal resistive index variation (IRRIV) test combined with eGFR can be included in a preoperative assessment to identify patients at high risk of developing AKI and accurately plan their management.

Postoperative acute kidney injury (PO-AKI) is a common complication after surgery associated with significant morbidity and mortality.^1–4^ Multiple renal stressors can induce perioperative kidney damage and thus PO-AKI, particularly in those highly susceptible patients who already present preoperatively inefficient mechanisms to maintain renal homeostasis.^5,6^ Intraoperative fluid restriction, kidney hypoperfusion, and congestion led by pneumoperitoneum^7^ may induce renal damage in high-susceptible patients undergoing prolonged robotic surgery in Trendelenburg position for oncologic pelvic disease. In addition, oncological patients are frequently exposed to nephrotoxic drugs, such as contrast media or nonsteroidal anti-inflammatory drugs (NSAIDs), that may contribute to kidney damage. Identifying patients at high risk of developing PO-AKI before surgery may help the physician to plan patients’ perioperative management in a more accurate way.^8^

Serum creatinine (sCr) is the standard of practice for quantifying preoperative renal function.^9^ Other tools have been proposed to identify highly susceptible patients, such as low preoperative estimated glomerular filtration rate (eGFR), and increase in cell cycle arrest and tubular damage biomarkers early during the postoperative care.^9^ However, preoperative sCr and eGFR alone are inadequate for assessing AKI risk before a kidney insult,^10^ and biomarkers cannot predict the risk of developing AKI before the injury occurs. In this setting, the assessment of renal functional reserve (RFR), ie, the capability of the kidney to increase GFR under physiologic or pathologic stress, may be more informative than sCr and eGFR alone. RFR is currently measured by a standardized protein loading test^11^ which is, however, cumbersome for routine clinical use. According to recent findings, RFR can be screened through a bedside ultrasound test: the intraparenchymal renal resistive index variation (IRRIV) test,^12^ which is noninvasive, rapid, and easy to perform at the patient’s bedside.

The rationale behind the IRRIV test is that a standardized external compression, leading to an increase in intraabdominal pressure and a decrease in renal perfusion, triggers the autoregulation of GFR via an afferent vasodilation. Afferent vasodilation is the common element shared by the protein loading and IRRIV tests, and can be assessed in a single patient by a drop in renal resistive index (RRI) in an interlobular artery. This delta-RRI correlates with RFR.^12^ IRRIV test has been validated in healthy individuals^13^ and previously investigated in scheduled cardiac surgery patients, but no information is currently available for other surgical specialities.^14^

This prospective observational study aims to preliminarily explore the predictive capability of the IRRIV test in identifying PO-AKI among patients with eGFR >60 mL/min/1.73m^2^ undergoing robotic abdominal surgery in Trendelenburg position for pelvic oncological disease.

## METHODS

This single-center, prospective, double-blinded, observational pilot study was registered before patient enrollment at clinicaltrials.gov (NCT04341974, principal investigator: Gianluca Villa, Careggi Hospital, date of registration: April 14, 2020) and was conducted in accordance with the STROBE guidelines outlined in the Declaration of Helsinki. The ethics committee of the Azienda Ospedaliero-Universitaria Careggi-University of Florence approved the study (CEAVC 13882; protocol number 2054/2019), and written informed consent was obtained from all the participants.

The selected patients underwent abdominal surgery at our hospital between September 2019 and March 2020. All patients aged >18 years undergoing robotic abdominal surgery with a planned duration of more than 4 hours and in Trendelenburg (−30°) position for pelvic oncological disease were considered for this study. Patients with a baseline eGFR<60 mL/min/1.73m^2^ (calculated at the preoperative outpatient evaluation using the 2009 Chronic Kidney Disease-Epidemiology Collaboration (CKD-EPI) equation^15^)were excluded, as well as those preoperatively treated with chemotherapy and those who underwent contrast media exposure within 7 days before surgery.

As a routine local practice, every patient underwent: (1) preoperative fasting limitation (2 hours for fluids), (2) preoperative interruption of nephrotoxic drugs, including NSAIDs and potentially nephrotoxic antibiotics for short-term prophylaxis (eg, aminoglycosides or glycopeptides), and avoidance of nephrotoxic drugs intraoperatively and postoperatively (within 3 days after surgery); perioperative avoidance of diuretics, except to treat volume overload (identified practically according with specific patient’s needs and in the absence of a specific definition), and (3) intraoperative hemodynamic monitoring and optimization. In every patient, a deep muscular blockage was maintained with rocuronium (with a target of train-of-four <1), and pneumoperitoneum induced via AirSeal was maintained with a median pressure value equal to 12 mm Hg. Intraoperative hemodynamic instability, defined as a variation of mean arterial pressure greater than 10% of the baseline value, bleeding (defined as a blood loss more significant than 200 mL), and abdominal pressure >12 mm Hg were dropout criteria.

### Baseline Kidney Function and Renal Adaptability to Increased Intraabdominal Pressure

Each patient underwent routine preoperative anesthesiologic and surgical assessments scheduled for major oncological abdominal surgery. Preoperative baseline renal function was evaluated in both terms of sCr and eGFR.

On the day of surgery, the IRRIV test was performed before the induction of anesthesia by a trained sonographer using a multi-frequency convex probe (Toshiba’s XarioTM 200, Doppler gate 2 to 4 mm, the lowest pulse repetition frequency without aliasing, the highest gain without obscuring background noise and the lowest wall filter^16^). First, the baseline RRI measurement was performed with the patient in the supine position after a rest of 5 minutes. Then, according to the results of the dose-response test,^12^ a fluid-bag 10% of patient’s body weight was applied to the abdominal wall for the renal stress testing, and the RRIs were recorded in a middle interlobular artery every minute for 10 minutes; the lowest of the 10 records was taken as the stress RRI. The method for measuring RRI and estimating Doppler-derived RFR has been described in detail by Samoni et al.^12^ According to previous data in the Literature, the IRRIV test was considered positive (presence of RFR) when the difference between the baseline RRI and stress RRI (delta-RRI) was ≥0.05.^12^ The physicians, including anesthesiologists who cared for patients in and out of the operating room, were blinded to these results.

**Table 1.** Characteristics of the Population at Baseline and Intraoperative Variables

Variable	Total (n = 53)	AKI (n = 8)	No AKI (n = 45)	OR [CI 95%]	*P*-value
Demographic variables					
Age (y)	68.9 [63.8–71.1]	69.3 [61.1–71.1]	68.8 [64.4–71.1]	1 [0.9–1.1]	.763
Male gender	45 (84.9%)	7 (15.6%)	38 (84.4%)	0.9 [0.1–6.8]	.921
Height (cm)	170 [167–176]	171.5 [167–180]	170 [167–176]	1.01 [0.9–1.1]	.812
Weight (kg)	74.6 ± 10.2	76 ± 12.3	74.4 ± 10	1.01 [0.9–1.1]	.714
BMI (kg/m^2^)	25.3 ± 2.3	25.5 ± 2.2	25.3 ± 2.4	1.04 [0.8–1.4]	.791
Comorbidities					
Variable	Tot (n = 53)	AKI (n = 8)	NO AKI (n = 45)	OR [CI 95%]	*P*-value
Vasculopathy	9 (16.7%)	2 (22.2%)	7 (77.8%)	1.8 [0.3–10]	.485
CAD	4 (7.5%)	0 (0%)	4 (100%)	0.6 [0.02–16.6]	.751
CHF	1 (1.9%)	0 (0%)	1 (100%)	1.9 [0.02–182]	.774
AH	30 (56.6%)	5 (16.7%)	25 (83.3%)	1.2 [0.3–5.4]	.785
DM	3 (5.7%)	1 (33.3%)	2 (66.7%)	2.6 [0.3–25.3]	.411
CHF	1 (1.9%)	0 (0%)	1 (100%)	1.9 [0.02–182]	.774
Preoperative variables					
Steroids	4 (7.5%)	1 (25%)	3 (75%)	2.6 [0.3–25.3]	.411
Immunosuppression	3 (5.7%)	1 (33.3%)	2 (66.7%)	3.7 [0.3–43.7]	.296
NSAIDs	10 (18.9%)	2 (20%)	8 (80%)	1.8 [0.3–10]	.485
ACEI	15 (28.3%)	0 (0%)	16 (100%)	0.1 [0.01–2.3]	.159
Sartans	8 (15.1%)	2 (25%)	6 (75%)	2.1 [0.4–11.9]	.391
β-blocker	10 (18.9%)	1 (10%)	9 (90%)	0.8 [0.1–6]	.854
sCr (mg/dL)	0.9 ± 0.1	1.1 ± 0.1	0.9 ± 0.1	11,569.4 [12.1–11,030,228]	.007*
eGFR (mL/min/1.73 m^2^)	82.2 [70.1–88.2]	66.9 [60.5–73.7]	83.9 [71.7–90]	0.9 [0.8–1]	.006*
Proteinuria + ++ +++	26 (49%) 26 (49%) 1 (2%)	3 (11.5%) 4 (15.4%) 1 (100%)	23 (88.5%) 22 (84.6%) 0 (0%)	1.4 [0.3–6.5] 21.5 [0.2–2257.3]	.670 .196
Intraoperative variables					
Duration of surgery (min)	215 [165–279.5]	268 [227.5–305]	197.5 [160–264]	1 [1–1.01]	.3495
Pneumoperitoneum duration (min)	178.5 [132.5–240]	239 [192.5–265]	168.5 [127.5–228.5]	1 [1–1.01]	.3005
Pneumoperitoneum pressure (mm Hg)	12.0 [12.0–12.0]	12.0 [12.0–12.0]	12.0 [12.0–12.0]	1.03 [0.51–2.08]	.9376
Fluid therapy (mL)	1375 [1000–1500]	1250 [875–1500]	1375 [1000–1500]	1 [1–1]	.7291
Blood loss (mL)	50 [50–100]	75 [50–150]	50 [50–100]	1 [0.99–1.01]	.5347
Diuresis (mL/kg/h)	0.66 [0.28–1.29]	0.40 [0.23–0.57]	0.82 [0.28–1.41]	0.23 [0.04–1.18]	.0776

Values were reported as median [25th–75th percentile], mean ± standard deviation or percentage (*P < .05).

Abbreviations: ACEI, angiotensin-converting enzyme inhibitor; AH, arterial hypertension; AKI, acute kidney injury; BMI, body mass index; CAD, coronary artery disease; CHF, chronic heart failure; CHeF, chronic hepatic failure (child-Pugh>2); CI, confidence interval; DM, diabetes mellitus; eGFR, estimated glomerular filtration rate; Immunosupp., immunosuppressor; OR, odds ratio; NSAIDs, nonsteroidal anti-inflammatory drugs; sCr, serum creatinine.

**Table 2.** Ultrasound preoperative renal parameters.

Variable	Total (n = 53)	AKI (n = 8)	No AKI (n = 45)	OR [CI 95%]	*P*-value
Tr. diam. (mm)	54.7 ± 7.8	57.5 ± 9	54.3 ± 7.6	1 [0.9–1.1]	.297
Long. diam. (mm)	103.7 [95.8–110.7]	105.6 [ −111.8103.3–111.8]	102.7 [93.8–110.7]	1 [0.9–1.1]	.427
Cortex (mm)	17.2 ± 3.4	16.3 ± 2	17.3 ± 3 6	0.9 [0.7–1.1]	.427
Medulla (mm)	35.6 ± 7.4	39.1 ± 9.3	35 ± 7	1 [0.9–1.2]	.170
delta-RRI	0. 055 ± 0.03	0. 026 ± 0.016	0. 061 ± 0.029	0.6 [0.4–0.9]	.007*
delta-RRI ≥0.05 delta-RRI <0.05	31 (58.5%) 22 (41.5%)	0 (0%) 8 (36.4%)	31 (100%) 14 (63.6%)	0.03 [0–0.5]	.017*

Values expressed as median [25th–75th percentile] or mean ± standard deviation and odds ratio (OR) with 95% confidence interval were reported (*P < .05).

Abbreviations: AKI, acute kidney injury; ATr. diam., transversal diameter; CI, confidence interval; delta-RRI, delta renal resistive index; Long. diam., longitudinal diameter; OR, odds ratio.

**Figure 1.**
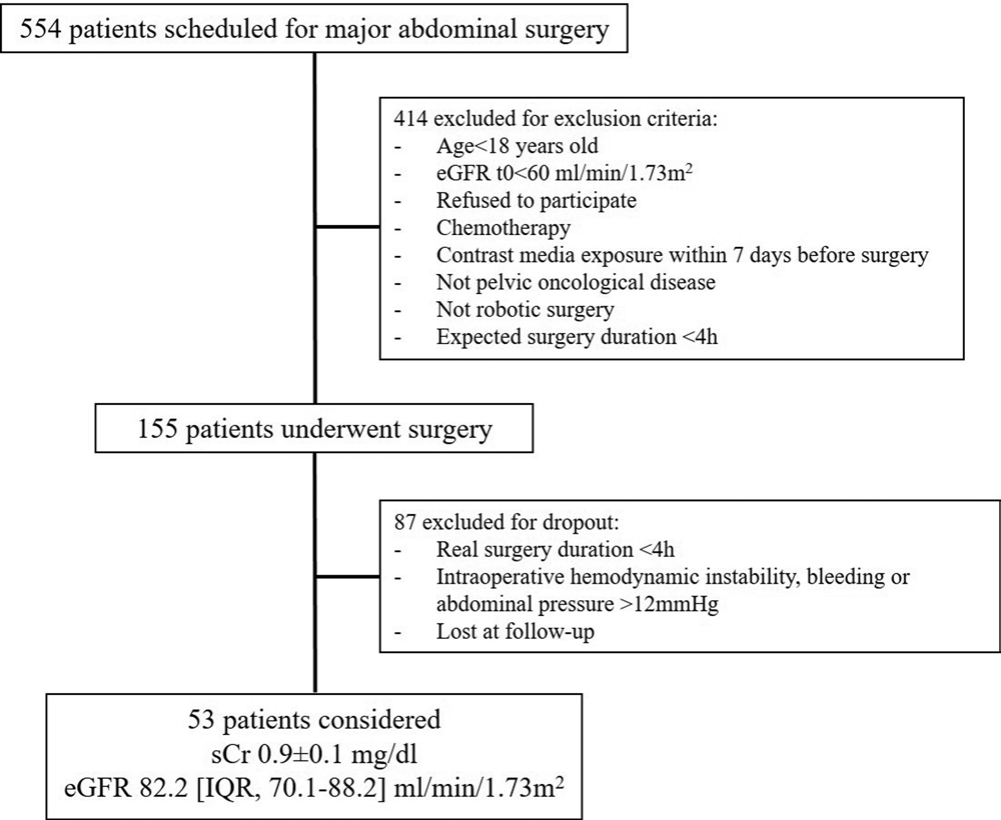
Flow chart of the study. eGFR indicates estimated glomerular filtration rate; sCr, serum creatinine.

**Figure 2.**
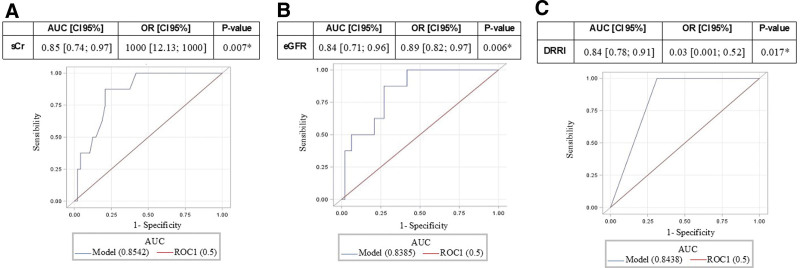
ROC-AUC for sCr (A), eGFR (B), and delta-RRI (C). Values are reported with 95% confidence interval (*P < .05). delta-RRI indicates delta renal resistive index; eGFR, estimated glomerular filtration rate; ROC-AUC, area under the receiver operating characteristic curve analysis; sCr, serum creatinine.

**Figure 3.**
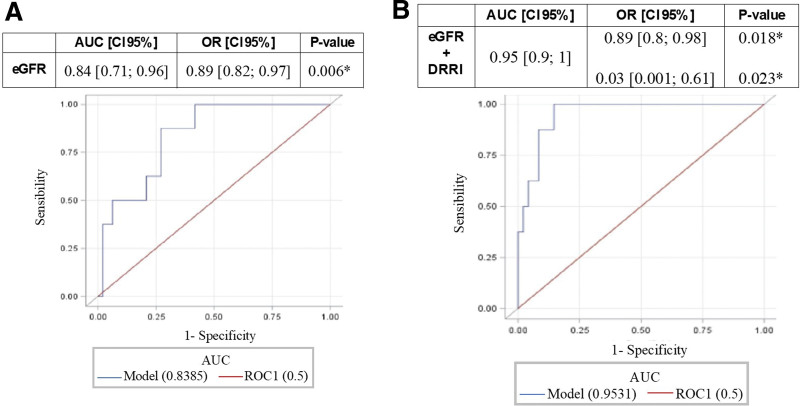
ROC-AUC for eGFR (A) and eGFR plus delta-RRI (B). Values are reported with 95% confidence interval (*P < .05). delta-RRI indicates delta renal resistive index; eGFR, estimated glomerular filtration rate; ROC-AUC, area under the receiver operating characteristic curve analysis.

SCr was postoperatively measured daily until hospital discharge. PO-AKI was diagnosed and graded according to the Kidney Disease Improving Global Outcomes (KDIGO) criteria^1^ within 72 hours postsurgery. Additional measures were length of hospitalization and kidney function at discharge and after 6 and 12 months.

### Statistical Analysis

Continuous parameters observed in the population are reported as median [25th–75th percentile] or mean ± standard deviation (SD), where appropriate; dichotomous parameters are expressed as raw number and percentage. The capability of Doppler-derived RFR in predicting PO-AKI was preliminarily explored with Receiver Operating Characteristic (ROC) curve analysis, and the Area Under the ROC Curve (AUC) and its 95% confidence interval (CI) were reported. A p-value of 0.05 was considered for statistical significance. Given the exploratory nature of the study and the lack of data in the literature, it is impossible to define a formal hypothesis and perform the power analysis for sample size calculation. An enrollment of 8 to 10 patients per month was expected, and a study duration of 6 months.

## RESULTS

Among the 554 patients scheduled for major abdominal surgery (Figure 1), 53 were enrolled for this pilot study. At the preoperative evaluation, the enrolled population presented a mean sCr of 0. 9 ± 0.1 mg/dl and an eGFR of 82.2 (95% CI, 70.1–88.2) mL/min/1.73 m^2^. The clinical characteristics of the enrolled population are highlighted in Table 1. The Doppler-derived RFR was obtained in every patient enrolled in this prospective study. Most of the enrolled patients were male patients undergoing robotic prostatectomy with lymph-node section (n = 32), or robotic radical cystectomy (n = 21). Cumulatively considering the first 3 postoperative days, 8 (15.1%) surgical patients developed PO-AKI (AKI group). PO-AKI reached the KDIGO stage 1 in most of the cases; only 1 patient developed an AKI KDIGO stage 2 at day 1 postsurgery. Mean surgery duration was 237 [IQR, 165–279] minutes, 265 for AKI, and 232 for no-AKI. Patients underwent intraoperative infusion of balanced crystalloid solutions (Ringer Acetate); median crystalloid infusion (mL/patients) was 1375 [IQR, 1000–1500]; 1250 [IQR, 875–1500] for AKI and 1375 [IQR, 1000–1500] for no-AKI patients. Median intraoperative mean arterial pressure was 80 [IQR, 75–86] mm Hg for AKI and 81 [IQR, 72–85] mm Hg for no-AKI patients. No patient required intraoperatively or postoperatively vasoactive infusion or blood components. Supplemental Digital Content 1, Table S1, http://links.lww.com/AA/E795 describes the overtime variation for sCr, fluid balance, urinary output, and diuretic use for both AKI and no-AKI patients. All the patients had maintained levels of sCr higher than the baseline for 4 (IQR, 3–5) postoperative days. Patients who developed PO-AKI had longer hospital stays than patients who did not. The mean baseline RRI was 0. 60 ± 0.02 (as normally distributed), for both AKI and no-AKI groups. As shown in Table 2, kidney morphometry by ultrasound did not significantly differ between groups. The mean preoperative delta-RRI value observed at the IRRIV test was 0. 055 ± 0.03. Thirty-one (58.5%) patients had delta-RRI ≥0.05 (suggesting adequate intrarenal myogenic adaptability to kidney hypoperfusion), while 22 (41.5%) patients had delta-RRI <0.05. The mean delta-RRI was 0. 026 ± 0.016 in the AKI group and 0. 061 ± 0.029 in the no-AKI group (*P* = .007). None of the patients with delta-RRI ≥0.05 developed PO-AKI; on the other hand, 8 (36.4%) patients with delta-RRI<0.05 developed PO-AKI. Sensitivity and specificity were 100% (95% CI, 63%–100%) and 68.8% (95% CI, 53.7%–81.3%), while positive (PPV) and negative predictive value (NPV) were 34.8% (95% CI, 16.4%–57.3%) and 100% (95% CI, 89.4%–100%). The ROC-AUC for PO-AKI prediction is described in Figures 2 and 3 for parameters as sCr, eGFR, and IRRIV test, alone or in combination. In particular, the capability to predict PO-AKI (expressed as ROC-AUC) was 0.85 (95% CI, 0.74–0.97, *P* = .007) for sCr, 0.84 (95% CI, 0.71–0.96, *P* = .006) for eGFR, 0.84 (95% CI, 0.78–0.91, *P* = .017) for delta-RRI (Figure 2). When combined with eGFR, the predictive capability for delta-RRI was increased (ROC-AUC 0.95 [95% CI, 0.9–1]; Figure 3).

### Outcomes

The mean length of the hospitalization was 5 ± 4.7 days, with a longer duration for the AKI (11. 3 ± 10.8 days) compared to the no-AKI group (6. 4 ± 5.3 days). At discharge, the last median sCr and eGFR were 0.9 [IQR, 0.7–1] and 90 [IQR, 82.6–93.8], respectively. In the AKI group, kidney function at hospital discharge was reduced (sCr 1.2 [IQR, 1.0–1.4] and eGFR 62.7 [IQR, 50.5–78.9]) when compared with those in the no-AKI group (sCr 0.8 [IQR, 0.7–0.9] and eGFR 90.8 [IQR, 84.8–94.9]). Consistently with this, at 6 and 12 months after hospital discharge, kidney function has been maintained in a normal range for the no-AKI group (sCr 0.9 [IQR, 0.8–1]/eGFR 88.8 [IQR, 80.1–96.8] and sCr 0.9 [IQR, 0.8–1]/eGFR 89.1 [IQR, 79–96] at 6 and 12 months, respectively), whereas it was reduced in the AKI group (sCr 1.4 [IQR, 1.4–1.5]/eGFR 57.8 [IQR, 45.1–60.3] and sCr 1.4 [IQR, 1.4–1.5]/58.2 [IQR, 43.9–61.1] at 6 and 12 months, respectively) (Supplemental Digital Content 2, Table S2, http://links.lww.com/AA/E796).

## DISCUSSION

In this prospective, double-blinded, observational pilot study, we preliminary demonstrate the capability of Doppler-derived RFR to predict PO-AKI when integrated within the standard of practice for patients with baseline eGFR ≥60 mL/min/1.73m^2^ undergoing prolonged robotic surgery in Trendelenburg position for pelvic oncological disease. Preoperative measurement of sCr is nowadays the standard of practice to quantify the preoperative patient’s renal function and risk for developing PO-AKI.^17^ eGFR has been proposed to better inform the physician on the baseline patient’s kidney function, also given the availability of valid data of the GFR measured in people of different races.^18^ Accordingly, in our study, both sCr and eGFR predict PO-AKI with a ROC-AUC of 0.85 (95% CI, 0.74–0.97, *P* = .007) and 0.84 (95% CI, 0.71–0.96, *P* = .006), respectively. It is well known that several variables highly influence both sCr and eGFR; moreover, these variables can assess instantaneous renal function at baseline, but have limited predictive value in predicting changes in renal function intraoperatively. A dynamic preoperative assessment was therefore proposed with tests exploring renal adaptability to assess the ability of the kidneys to readjust their function within a physiological range (ie, RFR). In our study, the kidney’s adaptive (myogenic) response to pneumoperitoneum was explored with the IRRIV test. In a prospective study, a preoperative oral high protein load was given as a physiologic stressor to assess renal capacity to increase GFR, measured by creatinine clearance (CrCl).^8^ The authors showed that a reduced preoperative RFR is highly predictive of postoperative AKI and could serve to identify those cases that could benefit from preventive measures or postoperative biomarker assessment. Besides the renal stress test performed with oral protein load, a bedside ultrasound stress test, ie, the IRRIV test, has been proposed as an easy, fast, cheap, and comfortable method to estimate the preoperative RFR.^12^ Our group previously explored the use of Doppler-derived assessment of RFR to predict AKI,^14^ confirming that the myogenic response aimed at modulating the intrarenal vascularization during transient kidney hypoperfusion is an essential component of RFR.^12^ We also found a significant correlation between IRRIV and protein load-induced CrCl variation (RFR),^13^ and recommended IRRIV as the simplest test for preoperative RFR measurement in cardiac surgery patients; more interesting, IRRIV was similar in this study to protein-loading RFR in predicting AKI or subclinical AKI.^14^ Our hypothesis was that the evaluation of this myogenic adaptability via a weight placed on the patient’s abdomen (to increase abdominal pressure and thus reduce kidney perfusion) may reproduce the same kidney vascular condition occurring during pneumoperitoneum for robotic abdominal surgery. The ability to decrease RRI during stress is interpreted as an indication of an active and healthy myogenic response. The hypothesis formulated by Villa et al. 2016 is that the decrease in renal perfusion should generate a dilation of afferent arterioles so that renal blood flow is maintained and RRI is decreased.^6^ An inability to vasodilate adequately and increase renal blood flow would thus be manifest as a low delta-RRI. In our study, we assessed the IRRIV test in the operating room just immediately before surgery. The patients’ baseline state explored in these conditions could have been quite different from their true baseline due to external stimuli that lead to renal arteries vasoconstriction or vasodilation (such as anxiety, elevated catecholamines, or dehydration). Nonetheless, being an overtime variation of RRI compared to the baseline, the concept of IRRIV test should not theoretically be affected by all these external stimuli that certainly influence RRI at the baseline (before the abdomen compression), but also during the entire 10-minute ultrasound assessment. In our study, the preoperative estimation of ultrasonographic assessment for RFR had a ROC-AUC equal to 0.84 (95% CI, 0.78–0.91, *P* = .017) in predicting PO-AKI. These results suggest that the pathophysiological clinical pathway of PO-AKI may be driven by hypotension and pneumoperitoneum potentially occurring in surgical patients undergoing robotic abdominal surgery.^7^ Thus, pneumoperitoneum was the main determinant capable of inducing renal hypoperfusion, and the preoperative IRRIV test can be considered to realistically predict how the patient’s kidneys will respond to a subsequent transient renal hypoperfusion.^19–21^ Interestingly, although the Doppler-derived RFR has a similar ability to parameters exploring the kidney function (ie, sCr and eGFR) in predicting PO-AKI, a combination of both seems to improve the capability in identifying patients at high risk for developing PO-AKI preoperatively. Particularly, for the combination of the IRRIV test and eGFR preoperative assessment, an ROC-AUC of 0.95 (95% CI, 0.9–1) has been reported in our study. Reasonably, the IRRIV test seems more informative when combined with eGFR than with sCr, being the former a more accurate and standardized sCr-based method for GFR estimation, normalized for patient’s anthropometric features. Unfortunately, we were not able to measure the precise increase in abdominal pressure induced by the standard stressor represented by the weight on the patients’ abdomen, nor to measure delta-RRI intraoperatively during pneumoperitoneum induction. These 2 aspects must be recognized as limitations of this study to design further prospective studies to assess delta-RRI intraoperatively (eg, by transoesophageal echocardiography or trans lumbar ultrasound).

Practically, the intention of proposing ultrasound assessment as the standard of practice in preoperative evaluation is to unveil patients with an eGFR identifying a normal renal function or CKD stage 1 to 2, according to KDIGO criteria,^22^ but who in fact already have a more severe renal impairment (with “normal or mildly reduced” eGFR but impaired renal myogenic response). Although training is needed for this ultrasound test, a simple unstructured training lasting about a couple of hours was sufficient in our experience to obtain a perfect correspondence between the IRRIV test performed by expert sonographers (who have conducted previous studies on healthy subjects and cardiac surgery patients) and those performed by new adopters.

Of interest, the significance of a delta-RRI in each individual patient resides in its negative predictive value. No patient with a delta-RRI ≥0.05 has ever manifested clinically significant AKI.

In patients with an eGFR >60 mL/min/1.73m^2^, in the absence of intraoperative hemodynamic instability or major intraoperative complications, a delta-RRI ≥0.05 has an acceptable negative predictive value to reasonably exclude PO-AKI. In patients with eGFR >60 mL/min/1.73 m^2^, but with impaired RFR assessed with a delta-RRI<0.05, a certain risk for PO-AKI development still exists and a further evaluation with biomarkers immediately after surgery could guide the physician to better stratify the risk of PO-AKI. The early identification of patients at high risk for AKI or with a subclinical AKI, through the measurements of biomarkers of kidney stress or injury, respectively, is mandatory as it seems that prompt interventions can change the course of AKI and improve patients’ outcomes.^23^ Consistently to the literature, in our study, PO-AKI was associated with reduced long-term renal function at 6 and 12 months postoperatively, although all patients had the same sCr level at hospital discharge observed at baseline. Unfortunately, we are not able to define a causative association between PO-AKI and a higher median level of sCr at 6 and 12 months postoperatively, considering the multiple confounding factors for kidney insults that characterize the postoperative period of oncological patients.

This study has some limitations. First, we were not able to cross-validate or externally validate our preliminary results. Furthermore, the oncological nature of the primary disease has been chosen among the inclusion criteria to make this relatively small cohort as much homogeneous as possible in terms of surgical characteristics (as duration or bloodshed). Our preliminary results can be applied only to this well selected and homogeneous population. Secondly, the method used for Doppler-derived RFR assessment is far from being generalizable, and certainly a more accurate method than a simple bag in the patient’s abdomen should be defined to ensure the correct weight application during the entire procedure. Certainly, a detailed analysis of the intrabdominal pressure led by external abdominal compression should be performed in the future; the lack of this information in the present study derives from ethical concerns about using urinary catheters or an intragastric tubes for pressure analysis in awake patients preoperatively. Third, we did not the chance to reassess Doppler-derived RFR at 6- and 12-month follow-up. The lack of information on the RFR consuming postoperatively is certainly a limitation of this study. Fourth, although not statistically significant, a longer duration of surgery was observed in the group with AKI and higher rates of preoperative proteinuria and long-term use of NSAIDs and Angiotensin-converting-enzyme inhibitors in the group without AKI, which must be investigated in the future. Finally, postoperative biomarkers of kidney damage and/or kidney stress should be assessed beside preoperative Doppler-derived RFR, and their effect in increasing PO-AKI prediction explored in larger population.

In conclusion, PO-AKI is a frequent complication in patients with eGFR >60 mL/min/1.73 m^2^ undergoing prolonged robotic surgery in the Trendelenburg position for pelvic cancer disease. Preoperative assessment of Doppler-derived RFR has a similar ability to sCr-based assessments (including eGFR measurement) in predicting PO-AKI. Nevertheless, similar to other cohorts, a combination of preoperative parameters exploring the kidney function and the kidney adaptability (IRRIV test) improves the capability in identifying patients at high risk for developing PO-AKI. The application of Doppler-derived RFR assessment is increasing in several contexts, and certainly larger studies that validate this application are required in different cohorts of surgical patients. E

## DISCLOSURES

**Name:** Gianluca Villa, MD.

**Contribution:** This author helped in the conception, design, and coordination of the study and drafting of the article.

**Name:** Sara Samoni, MD, PhD.

**Contribution:** This author helped in designing the study, reviewing the literature, and drafting the article.

**Name:** Mirko Muzzi, MD, PhD.

**Contribution:** This author helped in the conception, design, and coordination of the study and drafting of the article.

**Name:** Sergio Fabbri, PhD.

**Contribution:** This author helped in designing the study, reviewing the literature, and drafting the article.

**Name:** Faeq Husain-Syed, MD.

**Contribution:** This author helped in designing the study, reviewing the literature, and drafting the article.

**Name:** Marco Allinovi, MD.

**Contribution:** This author helped in designing the study, reviewing the literature, and drafting the article.

**Name:** Laura Paparella, MD.

**Contribution:** This author helped in designing the study, reviewing the literature, and drafting the article.

**Name:** Pietro Spatafora, MD.

**Contribution:** This author helped in designing the study, reviewing the literature, and drafting the article.

**Name:** Roberto Di Costanzo, MD.

**Contribution:** This author helped in designing the study, reviewing the literature, and drafting the article.

**Name:** Lorenzo Tofani, MD.

**Contribution:** This author helped in statistical analysis.

**Name:** Zaccaria Ricci, MD.

**Contribution:** This author helped in study design and article revision.

**Name:** Sergio Serni, MD.

**Contribution:** This author helped in study design and article revision.

**Name:** Stefano Romagnoli, MD, PhD.

**Contribution:** This author helped in the conception, design and coordination of the study, and drafting and the revision of the article.

**This manuscript was handled by:** Alexander Zarbock, MD.

## Supplementary Material


