# Supplementary material for: Doppler-Derived Renal Functional Reserve in the Prediction of Postoperative Acute Kidney Injury in Patients Undergoing Robotic Surgery
Source: Anesth Analg. 2024 Jun 17;139(1):211–9. doi: 10.1213/ANE.0000000000006967 (PMC11155286; doi:10.1213/ANE.0000000000006967)
Supplement: Supplementary file 1 [file ane-139-211-s001.docx]

| **Table S1** - Clinical parameters evaluated in the postoperative days 1-3. Values were reported as median [25^th^ - 75^th^ percentile], mean ± standard deviation, or percentage (*p<0.05). Abbreviations: AKI: acute kidney injury; sCr serum creatinine. The percentages in the second column are percentages of the total population (53%), while the last two columns are line percentages. | | | |
| --- | --- | --- | --- |
| **Variable** | **Total (n=53)** | **AKI (n=8)** | **No AKI (n=45)** |
| **sCr (mg/dL)**  Day 1  Day 2  Day 3 | 1 [0.8-1.1]  0.9 [0.8-1]  0.9 [0.8-1] | 1.6 [1.4-1.9]  1.4 [1.3-1.5]  1.3 [1.1-1.5] | 1 [0.8-1.1]  0.9 [0.7-1]  0.9 [0.7-1] |
| **Fluid balance (mL)**  Day 1  Day 2  Day 3 | 353 [40-667.5]  -35 [-310-529]  53.7 ± 503 | 395.5 [172- 505]  450 [-50-705]  159.4 ± 455 | 323 [40-689.5]  -177,5 [-360-380.5]  36.1±512.9 |
| **Urinary output (L)**  Day 1  Day 2  Day 3 | 2.9±0.8  2.7±1  2.4±0.6 | 3.5± 1.4  2.6±0.8  2.7±0.7 | 2.8±0.7  2.8±1  2.3±0.6 |
| **Diuretic (%)**  Day 1  Day 2  Day 3 | 2 (3.8%)  1 (1.9%)  1 (1.9%) | 0  0  0 | 2 (100%)  1 (100%)  1 (100%) |
