# Supplementary material for: Doppler-Derived Renal Functional Reserve in the Prediction of Postoperative Acute Kidney Injury in Patients Undergoing Robotic Surgery
Source: Anesth Analg. 2024 Jun 17;139(1):211–9. doi: 10.1213/ANE.0000000000006967 (PMC11155286; doi:10.1213/ANE.0000000000006967)
Supplement: Supplementary file 2 [file ane-139-211-s002.docx]

| **Table S2** - Length of the hospitalization and kidney function at discharge and after 6 and 12 months. Values were reported as median [25^th^–75^th^ percentile], mean ± standard deviation. Abbreviations: sCr serum creatinine; eGFR estimated glomerular filtration rate. | | | |
| --- | --- | --- | --- |
| **Variable** | **Total (n=53)** | **AKI (n=8)** | **No AKI (n=45)** |
| **Hospitalization (days)** | 5 ± 4.7 | 11.3 ± 10.8 | 6.4 ± 5.3 |
| **sCr (mg/dl)**  Discharge  6 months post-surgery  12 months post-surgery | 0.9 [0.7-1]  0.9 [0.8-1.1]  0.9 [0.9-1.1] | 1.2 [1.0-1.4]  1.4 [1.4-1.5]  1.4 [1.4-1.5] | 0.8 [0.7-0.9]  0.9 [0.8-1]  0.9 [0.8-1] |
| **eGFR (ml/min/1.73 m^2^)**  Discharge  6 months post-surgery  12 months post-surgery | 90 [82.6-93.8]  86.7 [77.3-94.9]  86.2 [68.1-94.3] | 62.7 [50.5-78.9]  57.8 [45.1-60.3]  58.2 [43.9-61.1] | 90.8 [84.8-94.9]  88.8 [80.1-96.8]  89.1 [79-96] |
